# Exploring the effects and interactions of meteorological factors on the incidence of scrub typhus in Ganzhou City, 2008–2021

**DOI:** 10.1186/s12889-023-17423-8

**Published:** 2024-01-02

**Authors:** Kailun Pan, Renfa Huang, Lingui Xu, Fen Lin

**Affiliations:** 1 School of Public Health and Health Management, Gannan Medical University, Jiangxi Province, Ganzhou, 341000 China; 2 Ganzhou Municipal Center for Disease Control and Prevention, Jiangxi Province, Ganzhou, 341000 China

**Keywords:** Scrub typhus, Meteorological factors, Distributed lag nonlinear model, Generalized additive model, Interactions

## Abstract

**Background:**

Scrub typhus poses a substantial risk to human life and wellbeing as it is transmitted by vectors. Although the correlation between climate and vector-borne diseases has been investigated, the impact of climate on scrub typhus remains inadequately comprehended. The objective of this study is to investigate the influence of meteorological conditions on the occurrence of scrub typhus in Ganzhou City, Jiangxi Province.

**Methods:**

From January 1, 2008 to December 31, 2021, we gathered weekly records of scrub typhus prevalence alongside meteorological data in Ganzhou city. In order to investigate the correlation between meteorological factors and scrub typhus incidence, we utilized distributional lag nonlinear models and generalized additive models for our analysis.

**Results:**

Between 2008 and 2021, a total of 5942 cases of scrub typhus were recorded in Ganzhou City. The number of females affected exceeded that of males, with a male-to-female ratio of 1:1.86. Based on the median values of these meteorological factors, the highest relative risk for scrub typhus occurrence was observed when the weekly average temperature reached 26 °C, the weekly average relative humidity was 75%, the weekly average sunshine duration lasted for 2 h, and the weekly mean wind speed measured 2 m/s. The respective relative risks for these factors were calculated as 3.816 (95% CI: 1.395–10.438), 1.107 (95% CI: 1.008–1.217), 2.063 (95% CI: 1.022–4.165), and 1.284 (95% CI: 1.01–1.632). Interaction analyses showed that the risk of scrub typhus infection in Ganzhou city escalates with higher weekly average temperature and sunshine duration.

**Conclusion:**

The findings of our investigation provide evidence of a correlation between environmental factors and the occurrence of scrub typhus. As a suggestion, utilizing environmental factors as early indicators could be recommended for initiating control measures and response strategies.

**Supplementary Information:**

The online version contains supplementary material available at 10.1186/s12889-023-17423-8.

## Introduction

Scrub typhus, caused by the bacterium Orientia tsutsugamushi (Ot), is an infectious zoonotic disease. Its clinical features include fever, charred scabs or ulcers, rashes, and enlarged lymph nodes, liver, and spleen. Failure to receive proper treatment can result in complications such as myocarditis and meningitis, and in severe instances, may even lead to mortality ([1] (pp. 2006–2014)). Due to the various serotypes of Orientia tsutsugamushi, there is a significant variation in the mortality rate. For individuals who are not treated promptly, the mortality rate ranges from 0 to 70% (with a median of 6%). Even after treatment, the patient's death rate can still be as high as 1.4%. The occurrence of scrub typhus presents a grave concern for public health in the country [2–4].

A substantial body of literature has demonstrated a significant correlation between meteorological factors, including temperature, precipitation, and hours of sunshine, and the occurrence and spread of infectious diseases [5–7]. The risk of scrub typhus infection is primarily influenced by meteorological factors through their impact on the activity of chiggers and hosts, as well as the likelihood of contact between chiggers and humans [8, 9]. In previous research regarding the link between scrub typhus and meteorological factors, the emphasis has predominantly been placed on the nonlinear nature of these factors. Nevertheless, little attention has been given to the delayed impacts of meteorological factors, and there has been a scarcity of quantitative investigations exploring the correlation between meteorological factors and the occurrence of scrub typhus [10, 11]. In addition, the impact of meteorological variables on the disease is influenced by variations in climatic conditions, socioeconomic conditions, and individual behaviors across different regions. Thus, the findings cannot be easily generalized to other regions due to these differences.

The incidence rate of scrub typhus in Jiangxi Province from 2006 to 2012 demonstrated a consistent upward trend [12]. Additionally, there were a total of eight provinces in mainland China that reported more than 5000 cases of scrub typhus between 2006 and 2018, with Jiangxi Province being one of them [13]. Since the first case was reported in Ganzhou City in 2006, the disease has been spreading, making it a high prevalence area for scrub typhus in Jiangxi Province [14]. Ganzhou City had the highest number of cases and incidence rate of scrub typhus in Jiangxi during the period of 2006–2017 [15]. Presently, instances of scrub typhus have been recorded in 11 prefecture-level cities within the province [15]. However, there has not been any prior reporting on the impact of meteorological elements on the occurrence of scrub typhus in Ganzhou City. Consequently, this study utilized the distributed lag nonlinear model(DLNM) and generalized additive model (GAM) to analyze the effects of weekly meteorological factors on the incidence of scrub typhus in Ganzhou City, Jiangxi Province. The objective was to provide a theoretical foundation for early warning prediction and epidemic prevention and control efforts.

## Material and methods

### Study area

**Fig. 1:**
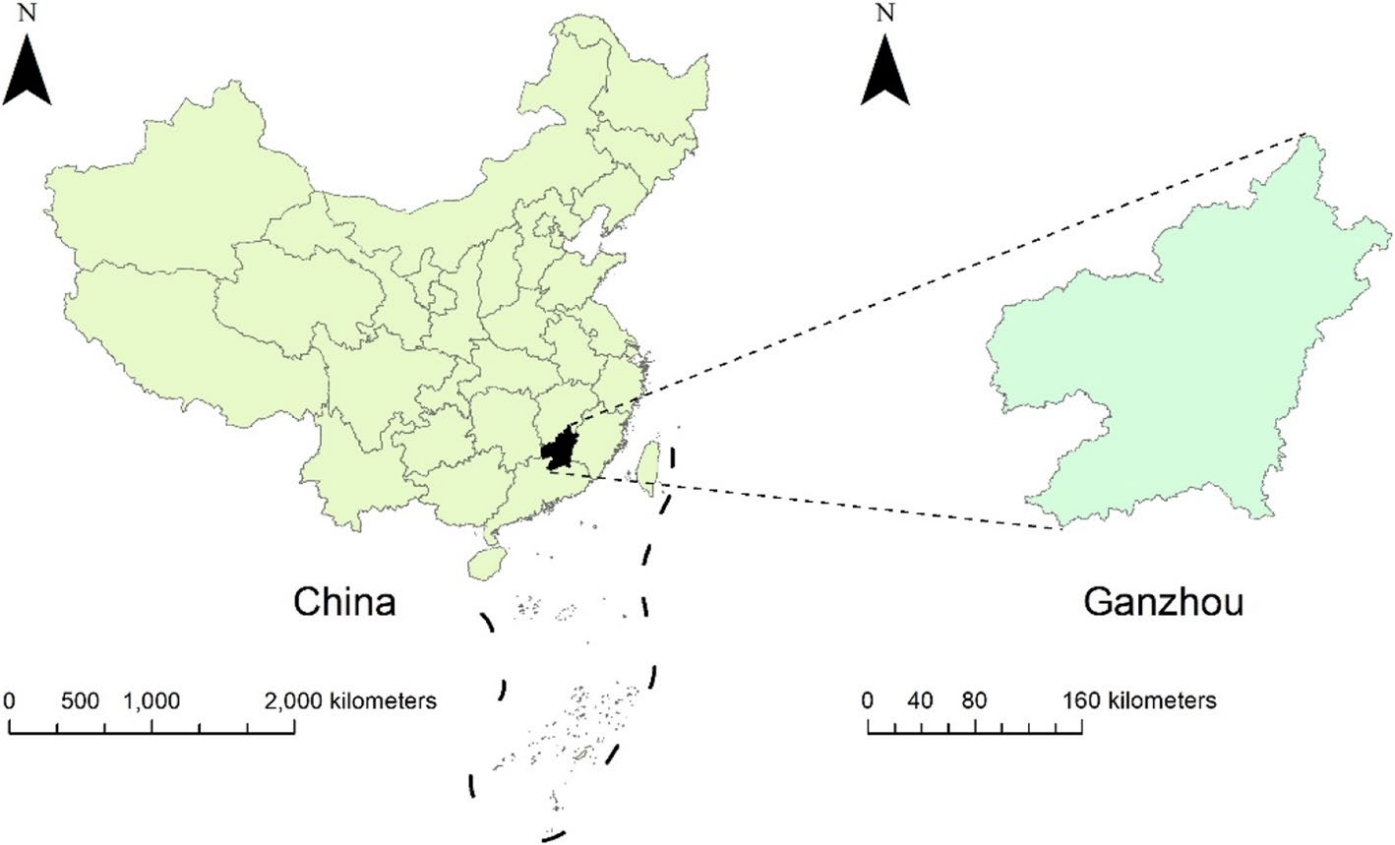
The placement of Ganzhou City in China's geographical region is depicted by the utilization of ArcGIS 10.8 for map creation. The foundation map utilized originates from the Data Center for Geoscience and Natural Resources Research, which is under the purview of the Chinese Academy of Sciences (CAS) (http://www.resdc.cn/)

Situated in the latitude range of 24°29′-27°09′N and the longitude range of 113°54′-116°38′E, Ganzhou City occupies a landmass of 39,379.64 square kilometers, representing approximately 23.6% of Jiangxi's total territory (Fig. 1). Positioned at the southern boundary of the central subtropical zone, Ganzhou City encounters a humid monsoon climate within its subtropical hilly and mountainous areas, featuring a notable concentration of rainfall during spring and summer seasons, accompanied by ample precipitation. As of the end of 2022, the city had a resident population of 8,988,100.

### Scrub typhus epidemic information

Data regarding scrub typhus cases were gathered from the National Disease Surveillance Information Management System (NDSIMS). The system compiled individual cases based on the date of onset and current location, along with details about the gender, profession, and age of the reported individuals. These data were collected from January 1, 2008 to December 31, 2021. The number of scrub typhus cases collected on a weekly basis during the study period remained in line with the specified diagnostic criteria (https://www.chinacdc.cn/).

### Meteorological information

We accessed the meteorological records for Ganzhou City during the corresponding timeframe from the National Meteorological Science Data Center (https://data.cma.cn/).These data include six meteorological variables: weekly average temperature (TEM), weekly average relative humidity (RHU), weekly average sunshine duration (SSD), weekly average wind speed (WIND), weekly average pressure (PRS), and weekly average precipitation (PRE).

### Statistical methods

#### Statistical description

We performed a descriptive analysis of scrub typhus cases and meteorological factors employing IBM SPSS Statistics 25.0. The association between the weekly count of cases and meteorological factors, along with the association amongst individual meteorological factors, was assessed via Spearman correlation analysis. To avoid multicollinearity, meteorological factors with correlation coefficients greater than 0.7 (|rs|> 0.7) were not included simultaneously in this study. In addition, to better address the problem of collinearity, SPSS 25.0 was used in this study to diagnose the data for collinearity, with variance inflation factor (VIF) < 10 and tolerance (Tol) > 0.1, indicating that the model does not suffer from the problem of collinearity.

#### Model building

The impact of meteorological factors on human health has a delayed effect and is generally non-linear. Distributed lag nonlinear model can solve this problem better by considering the exposure-lag response relationship on the basis of nonlinearity. Therefore, in this study, the use of DLNM enabled the analysis of the non-linear and lagged impacts of TEM, RHU, SSD, and WIND on the incidence risk of scrub typhus. Compared to the population of Ganzhou City, weekly new cases of scrub typhus infection in Ganzhou City are rare, and to avoid too much discretization of the data, this model used quasi-Poisson as a linking function, while incorporating influences such as time-seasonal trends and holiday effects to control confounding. When incorporating a meteorological factor in the function, the remaining meteorological factors are considered covariates (refer to the model below). The model is expressed as follows:

$$\mathit{log}\left(E\left({Y}_{t}\right)\right)=\alpha +cb\left(weather,df\right)+\sum ns\left({Z}_{i},df\right)+ns\left(time,df\right)+factor(holiday)$$

*E(Y*_*t*_*)*: expected number of scrub typhus cases at week t; *weather*: one of the four meteorological factors(TEM,SSD,WIND,RHU) associated with scrub typhus and screened by Spearman correlation analysis; *“Zi”* is a meteorological factor other than *weather*; *"time"* is employed for regulating seasonal and long-term patterns, whereas the *factor "holiday"* is a binary variable employed to regulate the impacts of holidays; the intercept of the model is denoted by *α*, while the cross-basis function *cb()* is utilized to depict the relationship between the number of scrub typhus cases and meteorological factors, considering exposure lags; additionally, the natural cubic spline function *ns()* is employed, with *"df"* representing the degree of freedom.

Significantly, this investigation utilized a generalized additive model to analyze the interplay between TEM, RHU, and SSD concerning the occurrence of scrub typhus. The fundamental model was:

$$\mathit{log}\left(E\left({Y}_{t}\right)\right)=\beta +s1\left(k,x\right)+s2\left(z\right)+factor(holiday)$$

*β* is the intercept; *k* is one of the meteorological factors (TEM, RHU, SSD), and *x、z* are the other meteorological factors. *s()* is the penalized spline function. *s1 (k, x)* is the spline function for the interaction of the variables *k* and *x*.

Taking into account the life cycle of chiggers and host animals, along with the incubation period of the disease, and building upon the findings of a prior investigation [1, 10], the utmost delay periods for temperature, relative humidity, duration of sunshine, and velocity of wind were established as 19, 17, 15, and 15 weeks respectively. To evaluate the model's robustness, we performed a sensitivity analysis by altering the range of degrees of freedom for the time variable (df = 6, 7, 8, 9/year). The selection of degrees of freedom and the identification of the optimal model were guided by the minimum principle of the Akaike Informativeness Criterion (AIC).Specifically, the degrees of freedom were 5, 3, 4, and 4 when TEM, WIND, RHU, and SSD were the independent variables, respectively, and the remaining meteorological factors were considered covariates. The model was created using R 4.2.3 software packages such as “mgcv”, “dlnm”, and others, with a test level of 0.05.

## Results

### Descriptive analysis of the incidence profile of scrub typhus and meteorological factors

 From January 1, 2008, to December 31, 2021, a total of 5,942 cases of scrub typhus were reported in Ganzhou City. The incidence rate showed an increasing trend from 2008 to 2018, with an average incidence rate of 3.56 per 100,000. However, from 2018 to 2021, there was a decreasing trend in the incidence rate, with an average incidence rate of 7.98 per 100,000. (Supplement Fig. 1) Among these cases, 34.9% (2,074 cases) were males and 65.1% (3,868 cases) were females, resulting in a male-to-female ratio of 1:1.86. In terms of case classification, the majority of cases were clinically diagnosed, accounting for 99.5% (5,910 cases). The onset of cases can be observed across all age groups, spanning from 6 months to 94 years. The majority of cases (59.2%) occur within the 25–60 year age bracket. Regarding occupation, the largest occurrences are documented within the farming, student, and retiree communities, with 5537, 112, and 111 instances, respectively.

The average weekly incidence of scrub typhus in Ganzhou City from 2008–2021 was 8 cases. The maximum number of cases recorded was 64, while the minimum was 0. The median number of cases was 2. The medians of WIND, PRE, PRS, TEM, SSD, and RHU were 1.09 m/s, 2.09 mm, 978.97 hPa, 20.87 °C, 4.16 h, and 80.57%, respectively (Table 1).

**Table 1:** Statistical description of weekly meteorological factors and the number of weekly scrub typhus cases in Ganzhou City, 2008–2021

	Mean	S.D	Min	P(_25_)	Median	P(_75_)	Max
WIND(m/s)	1.13	0.38	0.34	0.84	1.09	1.37	2.80
PRE(mm)	4.33	5.73	0	0.19	2.09	6.24	41.86
PRS (hPa)	979.03	6.12	963.54	973.93	978.97	984.13	992.04
TEM (℃)	19.63	6.76	1.89	14.14	20.87	26.09	29.53
SSD(h)	4.40	2.59	0	2.29	4.16	6.5	10.53
RHU (%)	79.33	8.20	43.29	74.86	80.57	84.86	96.86
weekly cases	8.13	12.05	0	0	2.00	12.00	64.00

*SD* Standard deviation

**Fig. 2:**
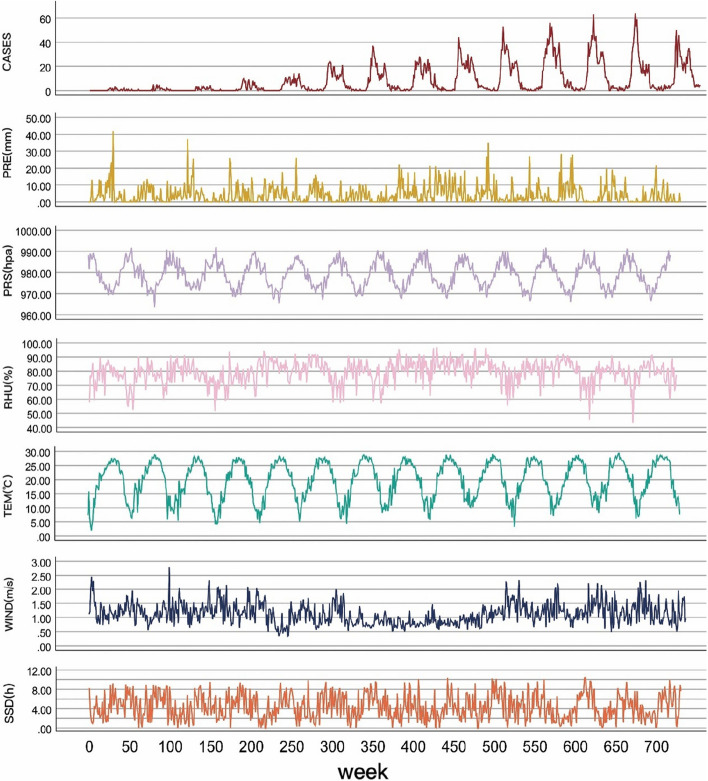
During the study period, a weekly time series plot depicting the correlation between the number of cases and meteorological factors was constructed

The manifestation of seasonal periodicity is apparent in the TEM and PRS, with opposite directions of cyclical fluctuations observed. Remarkably, there are prominent fluctuations in TEM preceding a substantial rise in the quantity of scrub typhus occurrences. Moreover, the peak in scrub typhus instances transpires following the temperature zenith. It is observable that the occurrences of scrub typhus display cyclical and seasonal patterns, with a delay (Fig. 2).

### Correlation analysis between weekly cases of scrub typhus and meteorological factors

To investigate the relationship between meteorological factors and weekly cases of scrub typhus in Ganzhou, a Spearman analysis was conducted in this study. The findings revealed positive correlations between scrub typhus cases, and TEM (rs = 0.605, *P* < 0.01), SSD (rs = 0.330, *P* < 0.01), and RHU (rs = 0.114, *P* < 0.01). Moreover, the cases of scrub typhus exhibited negative correlations with both WIND and PRS (rs = -0.153, -0.480, both *P* < 0.01). Nonetheless, no significant statistical association was observed between the cases of scrub typhus and PRE (*P* > 0.05). Furthermore, a strong correlation was observed between TEM and PRS (rs = -0.899).Therefore, the variables included in the distribution lag nonlinear model are TEM, SSD, RHU, and WIND (Table 2).

**Table 2:** Correlation matrix of weekly meteorological factors and weekly scrub typhus cases in Ganzhou, 2008–2021

	TEM	PRE	SSD	WIND	RHU	PRS	weekly cases
TEM	1						
PRE	0.238^**^	1					
SSD	0.376^**^	-0.403^**^	1				
WIND	-0.268^**^	-0.292^**^	0.011	1			
RHU	0.208^**^	0.641^**^	-0.592^**^	-0.428^**^	1		
PRS	-0.899^**^	-0.423^**^	-0.177^**^	0.315^**^	-0.366^**^	1	
weekly cases	0.605^**^	0.038	0.330^**^	-0.153^**^	0.114^**^	-0.480^**^	1

^**^P < 0.01

### Analysis of the distributional lag effect of meteorological factors on the incidence of scrub typhus disease

**Fig. 3:**
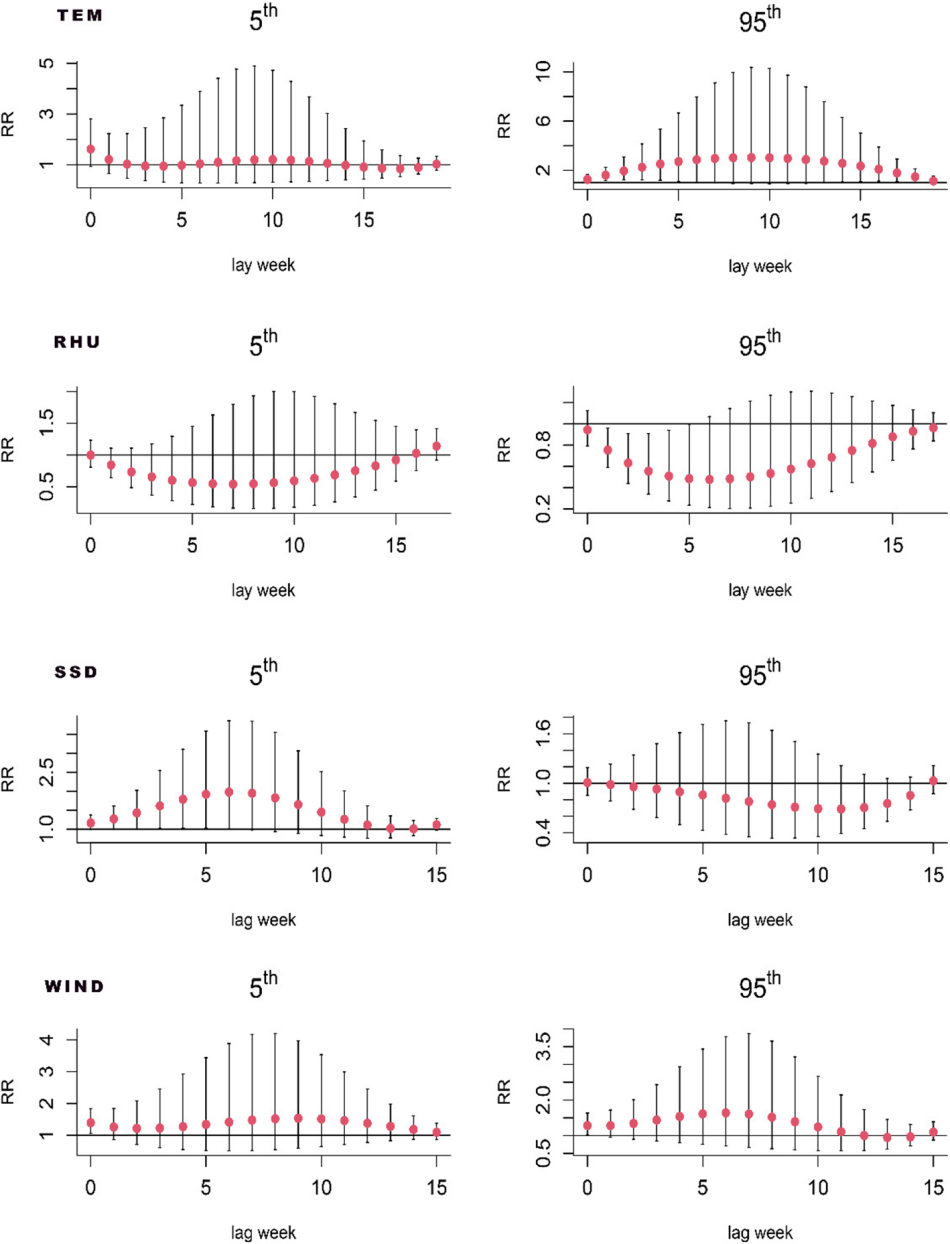
Lagged relationship between meteorological factors and the incidence of scrub typhus in the different lag week

For this investigation, we employed a distributional lag nonlinear model, utilizing the meteorological factors' median as a point of reference, and calculated the RR of scrub typhus infections and their 95% confidence intervals for the lagged weeks at the 95th percentile and the 5th percentile, respectively. As shown in Fig. 3, the relative risk(RR) was higher in high weather (95th), lagged week 5 (RR = 2.715, 95% CI:1.108–6.655) and week 6 (RR = 2.865, 95% CI:1.032–7.953), as well as in high relative humidity (95th), lagged week 4 (RR = 0.509, 95% CI. 0.275–0.94) and week 5 (RR = 0.484, 95% CI:0.235–0.996) had a lower risk of contracting scrub typhus; nevertheless, no statistically significant differences were observed at low temperatures (5th) as well as at low relative humidity (5th). The risk of scrub typhus infection was higher at lagged week 5 (RR = 1.924, 95% CI:1.033–3.584) and week 6 (RR = 1.981, 95% CI:1.017–3.859) under the short day length (5th); whereas, no statistically significant difference was observed on the long day length (95th). During the week of exposure, the relative risk reached higher values for both high (RR = 1.261, 95% CI:1.035–1.536) and low (RR = 1.22, 95% CI:1.015–1.467) wind speeds.

**Fig. 4:**
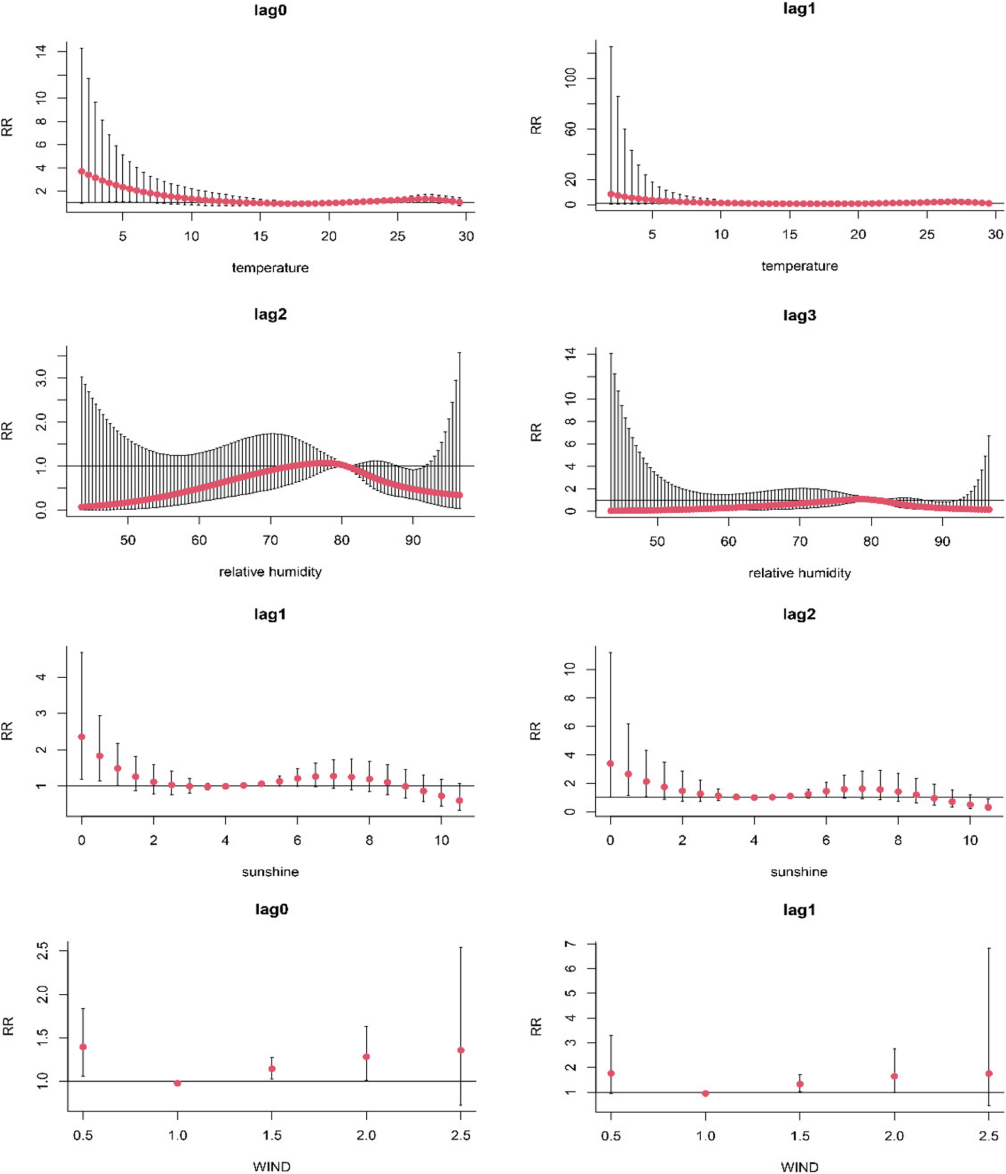
Lag-specific relationship of meteorological factors on the incidence of scrub typhus in the same lag week

Figure 4 presents specific associations between different meteorological factors and the incidence of scrub typhus during the same lag week. The risk of contracting scrub typhus was significantly higher when TEM was 3 °C (RR = 3.143, 95% CI:1.02–9.684), 5 °C (RR = 2.34, 95% CI:1.069–5.124), and 27 °C (RR = 1.319, 95% CI:1.017–1.71) during the week of exposure. A significant increase in RR was observed at week 1 of the lag when the weekly average temperature exceeded 26 °C. Furthermore, a significant RR was observed at RHU of 89–92% during both week 2 and week 3 of the lag, reducing the odds of contracting scrub typhus. Additionally, when SSD was less than 2 h, the chance of being infected with scrub typhus increased during both the first and second week of the lag. In the week of exposure, WIND of 0.5 m/s, 1.5 m/s, and 2 m/s were risk factors for the development of scrub typhus. Nevertheless, no significant RR values were found in the first week of the lag.

**Fig. 5:**
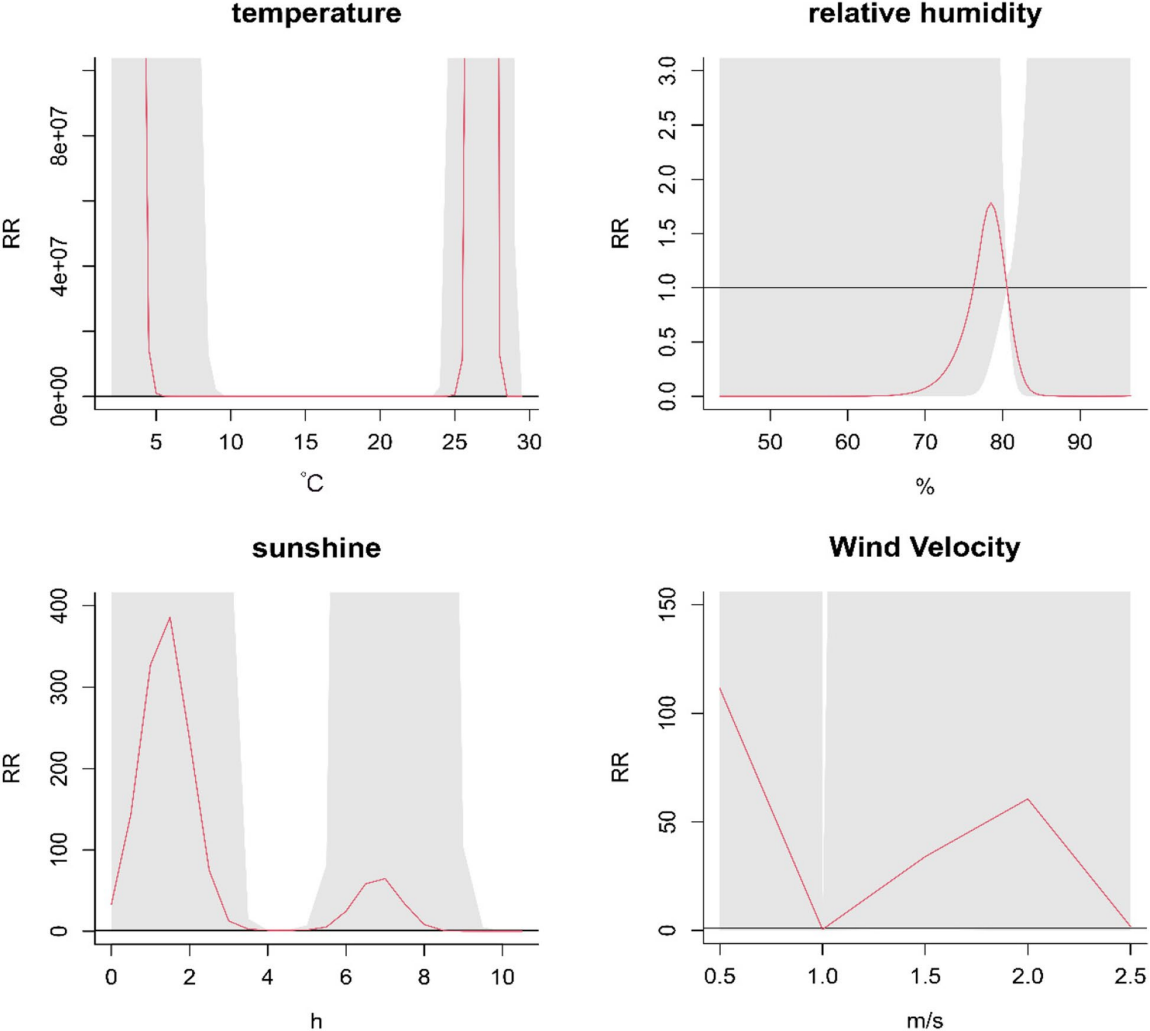
The relationship between meteorological factors and the overall response to the onset of scrub typhus

Figure 5 presents the comprehensive response of meteorological factors to the incidence of scrub typhus. The relationship between TEM and the incidence of scrub typhus follows a 'U' shaped non-linear exposure–response pattern. Similarly, the relationship between RHU and the incidence of scrub typhus exhibits an inverted 'V' shaped non-linear exposure–response pattern. Additionally, the relationship between SSD and the incidence of scrub typhus shows an 'M' shaped non-linear exposure–response pattern. Similarly, the relationship between WIND and the incidence of scrub typhus demonstrates an inverted 'N' shaped non-linear exposure–response pattern. The relative risks for TEM of 26 °C, RHU of 75%, SSD of 2 h, and WIND of 2 m/s reached their maximum values. The maximum values were 3.816 (95% CI: 1.395–10.438) for temperature, 1.107 (95% CI: 1.008–1.217) for relative humidity, 2.063 (95% CI: 1.022–4.165) for sunshine duration, and 1.284 (95% CI: 1.01–1.632) for wind speed.

### Interaction of meteorological factors on the incidence of scrub typhus

**Fig. 6:**
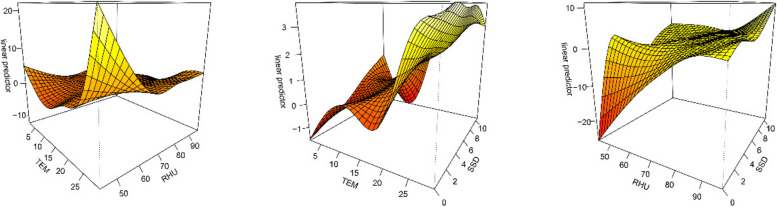
Interaction between meteorological factors and the incidence of scrub typhus in Ganzhou City, 2008–2021

In this study, a generalized additive model was utilized to analyze the interaction between TEM, RHU, and SSD in relation to the development of scrub typhus. The left side of Fig. 6 illustrates the interaction effect between RHU and TEM on scrub typhus. It shows that the risk of scrub typhus infection increases as air temperature increases and relative humidity decreases. The interaction effect of SSD and TEM on scrub typhus is depicted in the middle of Fig. 6. The risk of scrub typhus infection increases with higher air temperature and sunshine hours. The interaction effect of SSD and RHU on scrub typhus is depicted in the right side of Fig. 6. It is observed that the risk of scrub typhus infection increases as the relative humidity and sunshine duration increase. Therefore, the results of this study imply that the risk of scrub typhus infection in Ganzhou city escalates with higher TEM and SSD.

## Discussion

Using DLNM and GAM, our study examined the correlation between TEM, SSD, RHU, WIND, and the occurrence of scrub typhus in Ganzhou city during the period of 2008 to 2021. The results of the correlation analysis indicated that there is a positive correlation between the incidence of scrub typhus and TEM, SSD, and RHU. On the other hand, there is a negative correlation between the incidence of scrub typhus and WIND and PRS. These findings align with the existing literature [1, 10, 16, 17]. No statistical association was found between the incidence of the disease and weekly average precipitation. However, Kwak [18]concluded that there was a correlation between the incidence of scrub typhus and precipitation. This could be attributed to the variations in climates, types of chigger mites, and hosts among different regions, which can have varying impacts on the incidence of infectious diseases within each locality.

In this study, it was found through a descriptive study that the number of female cases of scrub typhus in Ganzhou City was greater than the number of male cases, and the occupations were mostly farmers, which aligns with the findings of Liao [19]. This is attributed to the fact that males of school-age work outside the home and females work in rural areas, and agricultural labor increases the risk of scrub typhus infection by increasing exposure to chiggers and lack of personal protection [20].

Scrub typhus is an acute febrile natural epidemic disease. While meteorological factors do not directly trigger the onset of scrub typhus, they can indirectly impact the disease by affecting the growth and development of host animals and chigger mites. Moreover, scrub typhus has a specific incubation period, indicating that the influence of meteorological factors on the disease may have a delayed effect. In this study, we analyzed the weekly lag effect of scrub typhus occurrence using DLNM and found that different climatic factors did not have the same lag effect on scrub typhus occurrence. These different lag times may be influenced by a variety of factors, including the growth and development of chiggers in the external environment, the tendency of humans to go outside, and seasonal changes in rodent populations [13].

In this study, we found that the risk of morbidity increased when TEM exceeded 26 °C. Temperature has an impact on the occurrence of scrub typhus by either inhibiting or promoting the activities of chiggers and hosts [21]. The optimal temperature range for the growth of chiggers is 20 °C-30°C. Among the chigger mites found in southern China and Southeast Asia, Leptotrombidium deliense is the most representative species [22]. It has been observed that the highest hatchability of field mites occurs when the temperature reaches 24 °C [13]. In addition, warmer environments are more conducive to farming and playing outside, thus increasing human exposure to chiggers.

The present study indicated that RHU of 89%-92% can decrease the risk of scrub typhus infection, which aligns with the findings of Zheng [23]. Zheng observed a decline in the incidence of scrub typhus with increasing relative humidity. However, previous research has demonstrated a higher disease risk in humid environments with larger populations of mites [24]. This phenomenon can be attributed to human interaction with the natural environment. For instance, during the dry season, more individuals tend to participate in outdoor activities, thereby increasing their exposure to chiggers [25].

In this study, we used DLNM to investigate the relationship between SSD and the incidence of scrub typhus. Our findings suggest that a weekly sunshine duration of less than 2 h is a risk factor for scrub typhus. Shorter durations of sunshine often coincide with rainfall, creating favorable conditions for rodents and chiggers [26]. Nevertheless, the results of the interaction in this study indicated that higher temperatures and longer hours of sunshine were risky conditions for the occurrence of scrub typhus, which is consistent with the results of a previous study [20]. Higher temperatures promote the growth and development of chiggers, while longer hours of sunshine increase the likelihood of human outings, thereby increasing the risk of scrub typhus infection [21, 27].

## Conclusion

To summarize, the risk of scrub typhus was found to have a nonlinear association with the weekly average temperature, weekly average relative humidity, weekly average sunshine duration, and weekly average wind speed, and there was a certain lag. Scrub typhus is more likely to appear in environments characterized by elevated temperatures and prolonged sunshine exposure. The findings of our investigation provide evidence of a correlation between environmental factors and the occurrence of scrub typhus. As a suggestion, utilizing environmental factors as early indicators could be recommended for initiating control measures and response strategies.

### Strengths

In this study, we choose to analyze time in weeks instead of months in order to obtain a more accurate range of lag for different levels of exposure. Secondly, this study uses three methods (correlation coefficient, variance inflation factor, and tolerance) to jointly determine the multicollinearity of the independent variables, which can further reduce the possibility of collinearity. Additionally, this study is the first to use a mathematical model to quantitatively analyze the relationship between climatic variables and scrub typhus in the region. While previous studies have only examined the delayed effect of meteorological factors, our study goes further by analyzing both the delayed effect of a single meteorological factor on the onset of scrub typhus and the interaction of two meteorological factors on the onset of scrub typhus. We employed a distributional lag nonlinear model and a generalized additive model to accomplish this, and considered four different meteorological factors to characterize the exposure-lag relationship and its interaction.

### Limitations

This study also has certain limitations and shortcomings. First of all, due to insufficient attention to scrub typhus and the inability to correctly diagnose scrub typhus, resulting in underreporting and misreporting of scrub typhus. Secondly, due to the difficulty of collecting lag week data for a specific meteorological factor, this study did not directly use lag weeks for GAM analyses. Thirdly, the weekly farming intensity of farmers, which is a key occupation associated with morbidity, impacts their exposure to chiggers and consequently their likelihood of contracting chiggers. However, acquiring accurate data on weekly farming intensity is arduous and the reliability of the data cannot be ensured. Finally, scrub typhus is a complex disease influenced by various factors including climate, environment, socioeconomic factors, host density, and vector mite density. Therefore, to better understand the factors influencing cases of scrub typhus, additional research is required on various aspects. This includes investigating natural environmental factors, socioeconomic factors such as agricultural activities and human activities, and individual behavioral factors.

### Supplementary Information

**Additional file 1: S figure 1.** Overall trend of scrub typhus cases in Ganzhou City, 2008-2021.

**Additional file 2. **Supplementary information.

## Data Availability

All data generated or analysed during this study are included in the Supplementary information files.

## Abbreviations

Ot: Orientia tsutsugamushi
TEM: Weekly average temperature
RHU: Weekly average relative humidity
SSD: Weekly average sunshine duration
WIND: Weekly average wind speed
PRS: Weekly average pressure
PRE: Weekly average precipitation
