# Supplementary figures and images for: Exploring the effects and interactions of meteorological factors on the incidence of scrub typhus in Ganzhou City, 2008–2021
Source: BMC Public Health. 2024 Jan 2;24:36. doi: 10.1186/s12889-023-17423-8 (PMC10763082; doi:10.1186/s12889-023-17423-8)

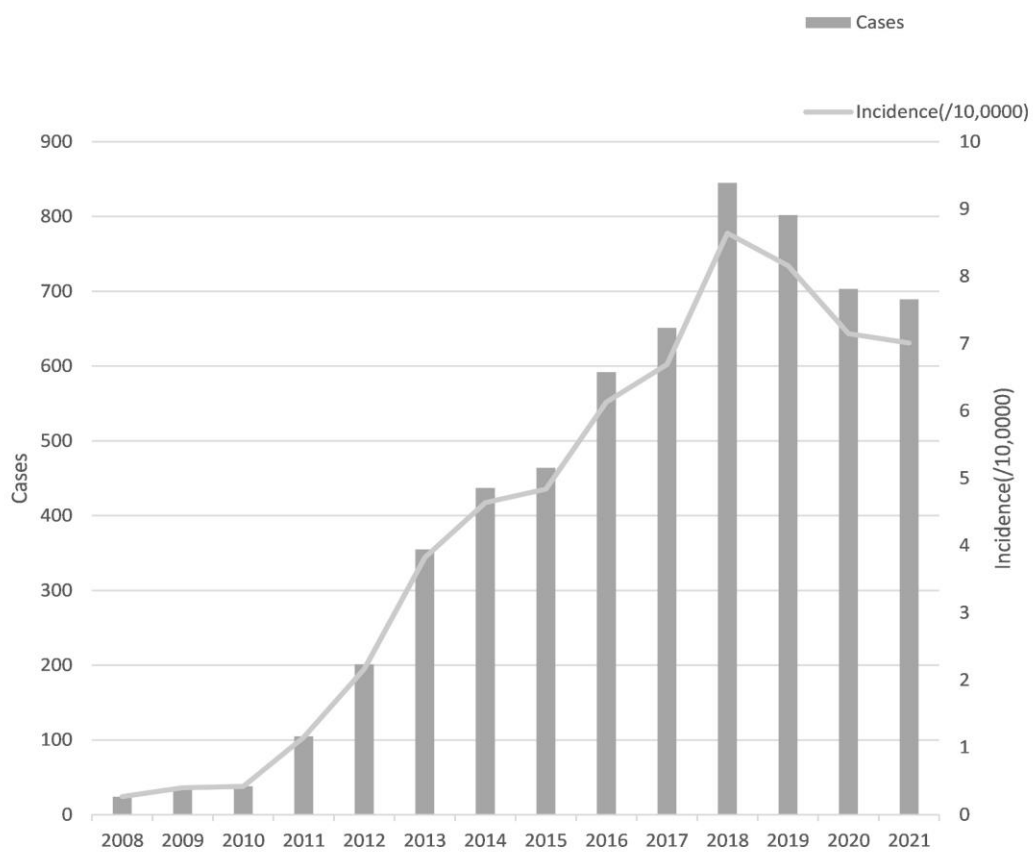

S figure 1-"Overall trend of scrub typhus cases in Ganzhou City, 2008-2021"

Supplement: Supplementary file 1 — Additional file 1: S figure 1. Overall trend of scrub typhus cases in Ganzhou City, 2008-2021. [file 12889_2023_17423_MOESM1_ESM.pdf]
